# Prolonged fasting and glucocorticoid exposure drive dynamic DNA methylation in northern elephant seals

**DOI:** 10.1242/jeb.250046

**Published:** 2025-07-25

**Authors:** Emily F. Gibson, Julia María Torres-Velarde, David C. Ensminger, Diana D. Moreno-Santillán, Daniel E. Crocker, José Pablo Vázquez-Medina

**Affiliations:** ^1^ Department of Integrative Biology, University of California, Berkeley, 3040 Valley Life Sciences Building #3140, Berkeley, CA 94720-3140, USA; ^2^ Department of Biology, Sonoma State University, Rohnert Park, CA 94928, USA

**Keywords:** Epigenetics, Bisulfite sequencing, Marine mammals

## Abstract

Northern elephant seals experience prolonged fasting while breeding, molting and undergoing postnatal development. Fasting elephant seals adjust neuroendocrine function and gene expression to cope with potentially detrimental effects associated with extended fasting. DNA methylation alters gene expression by modulating accessibility to regions necessary to initiate transcription. The effect of fasting and glucocorticoids on DNA methylation in elephant seals is understudied. We evaluated whether fasting alters global blood DNA methylation, the potential correlation between increased glucocorticoids and methylation, and the effects of glucocorticoids on DNA methylation in cultured northern elephant seal muscle cells. We found that fasting transiently increases blood DNA methylation and that blood DNA methylation levels correlate with plasma cortisol. We then conducted bioinformatic analyses to identify regions in the northern elephant seal glucocorticoid receptor (GR) promoter that influence gene transcription through methylation (CpG islands). We identified one CpG island within the putative GR promoter. Methylation in this region, however, was unaffected by prolonged fasting. We then investigated whether exogenous glucocorticoids alter DNA methylation and gene expression profiles in seal muscle cells in primary culture (myotubes). Exposure to glucocorticoids for 12 or 48 h decreased DNA methylation while upregulating pro-survival gene expression in northern elephant seal muscle cells. Our results show that whereas prolonged fasting transiently increases DNA methylation in northern elephant seal blood, sustained exposure to exogenous glucocorticoids decreases DNA methylation and activates a pro-survival transcriptional program in seal muscle cells. Therefore, our results suggest that DNA methylation is a plastic, potentially cell type-specific response that regulates gene expression in fasting northern elephant seals.

## Teaser

**Summary:** DNA methylation is a plastic response to fasting and increased glucocorticoids in northern elephant seals, highlighting the importance of epigenetic regulation of gene expression during energetically demanding conditions.

## INTRODUCTION

Northern elephant seals (*Mirounga angustirostris*) spend most of their life feeding at sea, returning to land to breed and molt (Le Boeuf and Laws, 1994). While on land, elephant seals naturally experience prolonged periods of absolute food and water deprivation (fasting) (Champagne et al., 2012a). Elephant seal pups nurse for a month before fasting for 2 months while undergoing postnatal development (Le Boeuf and Laws, 1994). Prolonged fasting induces changes in gene expression and neuroendocrine function that likely help elephant seals cope with potentially detrimental effects associated with extended food and water deprivation, such as oxidant stress and inflammation (Ensminger et al., 2021; Torres-Velarde et al., 2021; Vázquez-Medina et al., 2010).

DNA methylation is a biochemical mechanism that regulates gene expression by reversibly adding a methyl (CH_3_) group to a nitrogenous base in the DNA sequence of promoter regions (Dudley et al., 2011; Jeltsch, 2006). CpG islands are the most common methylation areas in gene promoters (Bird, 1986; Gardiner-Garden and Frommer, 1987; Holliday and Pugh, 1975). Generally, DNA methylation suppresses gene expression as a result of the methyl groups interfering with protein–DNA and DNA–DNA interactions that activate transcription (Boyes and Bird, 1992). As DNA methylation is a reversible biological signal that can function as an on–off switch to control gene expression (Ramchandani et al., 1999), it represents a conserved strategy to quickly respond to changing environmental conditions.

Studies in true hibernators such as thirteen-lined ground squirrels and Syrian hamsters, torpid bats and brumating Chinese alligators suggest that DNA methylation is an essential mechanism for regulating gene expression in response to changing environmental conditions (Alvarado et al., 2015; Coussement et al., 2023; Lin et al., 2020; Liu et al., 2023; Srere et al., 1992). However, the effects of fasting on DNA methylation in marine mammals have not been previously examined. In elephant seals, prolonged fasting increases glucocorticoid release (Ortiz et al., 2003, 2001), and previous work shows that glucocorticoid receptor (GR) signaling is crucial to the elephant seal's response to fasting (Avalos et al., 2023; Ensminger et al., 2021). In elephant seal muscle cells in primary culture, GR signaling induced by the synthetic glucocorticoid dexamethasone downregulates pro-inflammatory genes while upregulating genes that drive metabolic adjustments to support cell survival during energetic stress (Torres-Velarde et al., 2021).

Here, we studied global blood DNA methylation in fasting northern elephant seals and cultured northern elephant seal muscle cells treated with exogenous glucocorticoids. We also analyzed the northern elephant seal GR promoter region for specific methylation patterns. We found that global blood DNA methylation increases reversibly with prolonged fasting, in concert with plasma cortisol levels in northern elephant seal pups. In contrast, sustained exposure to dexamethasone decreased DNA methylation while upregulating pro-survival genes in northern elephant seal muscle cells in primary culture. Hence, our results suggest that DNA methylation is a plastic, dynamic response that likely contributes to regulating gene expression in simultaneously fasting and developing seals. Our results also suggest that changes in DNA methylation in response to increased glucocorticoid treatment might be tissue and gene specific.

## MATERIALS AND METHODS

### Animal handling and sample collection

**Fig. 1.**
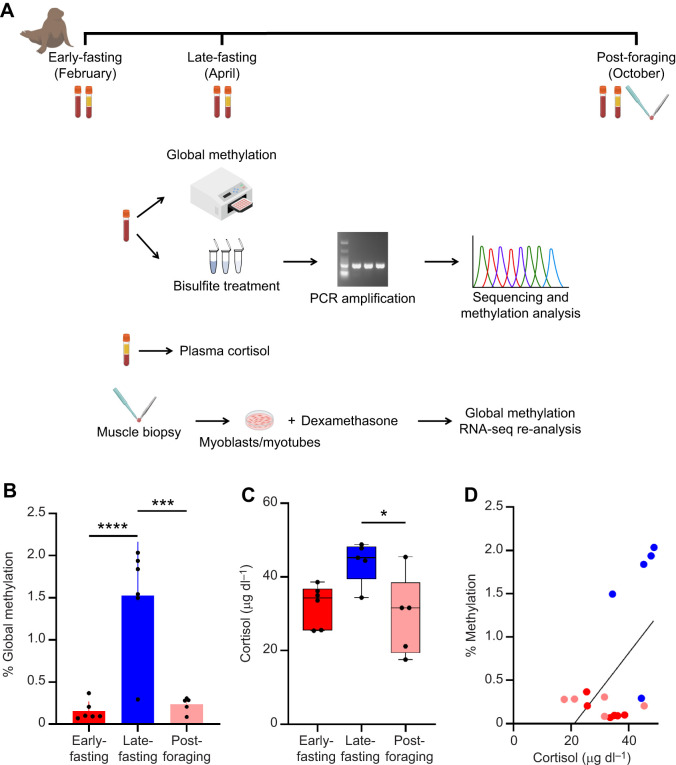
**Fasting increases blood DNA methylation in northern elephant seals.** (A) Sampling protocol (see Materials and Methods for details). (B) Blood DNA methylation in northern elephant seals during early-fasting, late-fasting and post-foraging periods. (C) Plasma cortisol concentrations in early-fasting, late-fasting and post-foraging animals. Boxes extend from the 25th to 75th percentiles, whiskers show the minimum and maximum values, and the lines show the median. Data are means±s.d. Asterisks indicate significance (Kruskal–Wallis in B, ANOVA in C; *P<0.05, ***P<0.001, ****P<0.0001. (D) Correlation of plasma cortisol and global blood DNA methylation; Deming linear regression model: *y*=13.82*x−*4.25, 95% confidence interval intercept −7.429 to −1.075, slope 5.674 to 21.97 (*r*=0.554, *P*=0.0255). Circles represent individual animals.

Animal work was approved by Sonoma State University and UC Berkeley Institutional Animal Care and Use Committees. Samples were collected under National Marine Fisheries Service Permit (NMFS) no. 1908 (PI Dan Costa, UCSC). Primary cells were derived under NMFS authorization no. 24479. Northern elephant seals, *Mirounga angustirostris* (Gill 1866), were sampled during three time periods from February to October at Año Nuevo Reserve (San Mateo County, CA, USA). The first sampling corresponded to early-fasting seal pups (<1 week post-weaning, unmolted animals, male and female, *N*=6). The second sampling corresponded to late-fasting pups (∼6 weeks post-weaning, male and female fully molted animals, *N*=6). The third sampling corresponded to post-foraging animals (∼8 months old, male and female, *N*=5) returning from their first oceanic feeding trip (Fig. 1A). Animals were immobilized with tiletamine/zolazepam and ketamine as previously described (Vázquez-Medina et al., 2010). Blood samples were collected from the extradural vein into chilled EDTA vacutainers (BD Biosciences, San Jose, CA, USA) and transported on ice to UC Berkeley. Plasma was separated by centrifugation, and the white blood cell layer was aliquoted and frozen at −80°C until DNA methylation analysis. Muscle biopsies of animals returning from their first oceanic feeding trip were collected from the longissimus dorsi in the posterior flank using a 6 mm biopsy needle (Vázquez-Medina et al., 2010), rinsed with Hanks' Balanced Salt Solution (Gibco, Waltham, MA, USA), stored in cold Ham's F10 nutrient mix (Gibco) supplemented with antibiotics, and transported on ice to UC Berkeley for cell isolation (Torres-Velarde et al., 2021).

### DNA extraction, global DNA methylation and cortisol measurements

Genomic DNA was extracted using the salting method (Miller et al., 1988). Samples were incubated with lysis buffer (5 mol l^−1^ NaCl, 1 mol l^−1^ Tris, 0.5 mol l^−1^ EDTA, 10% sodium dodecyl sulfate; Invitrogen, Waltham, MA, USA) and proteinase K (Promega, Madison, WI, USA) overnight at 55°C. DNA was precipitated with 100% ethanol, washed twice with 70% ethanol, and resuspended in Tris-EDTA (Invitrogen). DNA was purified using a Wizard^®^ SV Gel and PCR Clean-Up System kit (Promega) and quantified using a NanoDrop spectrophotometer (Nanodrop Technologies, Wilmington, DE, USA) and a Qubit fluorometer (Invitrogen). Percentage global DNA methylation was quantified in triplicate (coefficient of variation, CV=15.2) using 100 ng of DNA and a colorimetric Global DNA Methylation Assay kit (Abcam, Cambridge, UK). Plasma cortisol (CV=6.9) was quantified in 20 µl of plasma diluted 1:2 using an enzyme-linked immunosorbent assay (ELISA; Alpco, Salem, NH, USA) previously validated for elephant seals (McCormley et al., 2018).

### Promoter identification and characterization

The northern elephant seal GR gene was identified based on a sequence similarity search by comparing the database of annotated sequences in the elephant seal genome (Torres-Velarde et al., 2021) with the human GR sequence (UniProt: P04150). The similarity search was conducted using DIAMOND (double index alignment of next-generation sequencing data) under the ‘more-sensitive’ parameter (Buchfink et al., 2015). Corresponding promoter sequences were identified upstream of the elephant seal GR gene by aligning the elephant seal sequences with publicly available marine mammal sequences using BLAST (https://blast.ncbi.nlm.nih.gov). Once the putative promoter region was identified, 3341 base pairs upstream of the start codon of the GR gene, including the 5′ untranslated region, were used to identify CpG islands with MethPrimer (Li and Dahiya, 2002; http://www.urogene.org/cgi-bin/methprimer/methprimer.cgi). Transcription factor binding sites were identified within the CpG islands using Nsite (http://www.softberry.com/berry.phtml?topic=nsite&group=programs&subgroup=promoter). The transcription start site (TSS) was identified using TSSG software (Shahmuradov and Solovyev, 2015; Solovyev et al., 2010). Only regions with 0 mismatch between the elephant seal genome and the transcription factor binding site were considered for further analysis.

### Bisulfite conversion, PCR amplification, Sanger sequencing and methylation analysis

Complete deamination of unmethylated cytosine residues to uracil was carried out with 1 μg genomic DNA and sodium bisulfite, using the Qiagen EpiTect Bisulfite Kit (Qiagen, Hilden, Germany). The concentration of bisulfite-converted DNA was measured using a Nanodrop spectrophotometer (Nanodrop Technologies). Bisulfite-sequencing PCR (BSP) primers were designed using the MethPrimer tool (Li and Dahiya, 2002; https://www.methprimer.com/cgi-bin/methprimer/methprimer.cgi) to amplify fragments of about 300 base pair (bp) and synthesized by Integrated DNA Technologies (Coralville, IA, USA). The GR BSP1 primers were designed to amplify a region in the CpG island 259 base pairs long, which contained 56 CpG sites. The GR BSP2 primers were designed to amplify a region in the CpG island 339 base pairs long, with 66 CpG sites. The GR BSP3 primers were designed to amplify a region in the CpG island about 288 base pairs long, which contained 28 CpG sites. Primer sequences are listed in Table 1. PCR fragments were amplified using Platinum Taq DNA Polymerase (Thermo Fisher Scientific, Waltham, MA, USA), purified using the Wizard^®^ SV Gel and PCR Clean-Up System (Promega) and verified on 2% agarose gels. Amplified fragments were Sanger-sequenced at the UC Berkeley DNA sequencing facility. Sequences were analyzed using the Quantification tool for Methylation Analysis (QUMA, http://quma.cdb.riken.jp/) (Kumaki et al., 2008). To assess bisulfite conversion efficiency and determine CpG site methylation frequency, only sequences with a non-CpG cytosine conversion rate of ≥90% were retained for analysis. For each primer set (GR BSP1, BSP2 and BSP3), a total of 16 amplicons were obtained and sequenced in both forward and reverse directions, resulting in 32 sequences per primer. Sequences were derived from 5–6 biological replicates per condition. Following quality control, four sequences from BSP1, two from BSP2 and two from BSP3 were excluded from downstream analyses.

**Table 1.** Primers used to amplify and sequence regions in the CpG island of the northern elephant seal glucocorticoid receptor (GR) promoter

Primer name	Sequence	bp	*T*_m_
MA-GR-BSP-1 (F)	TTT GTT AGA GGT AAG AGG TGG G	22	57.4°C
MA-GR-BSP-1 (R)	AAA ACT CAA ATT CCT CCC CC	20	59.3°C
MA-GR-BSP-2 (F)	GGG GGA GGA ATT TGA GTT TT	20	59.3°C
MA-GR-BSP-2 (R)	ACT ACA AAC TAT CAA CCC CCT A	22	54.6°C
MA-GR-BSP-3 (F)	GAG AAA GAA TTT AAT AGG TTT GGA TA	26	55.8°C
MA-GR-BSP-3 (R)	TTA CAA ATA ACT ATC ACA TTA ATA AAC C	28	54.0°C

bp, base pairs; *T*_m_, melting temperature.

### Primary cell isolation, differentiation and dexamethasone treatment

Muscle progenitor cells were isolated from muscle biopsies and characterized as described in our previous work (Torres-Velarde et al., 2021). Cells derived from three post-foraging animals were seeded in tissue culture dishes coated with 0.01% collagen (Sigma, St Louis, MO, USA) in Ham's F10 nutrient mix (Gibco) supplemented with 20% fetal bovine serum (Seradigm, Radnor, PA, USA), 100 mmol l^−1^ Hepes (Gibco) and 1% Antibiotic-Antimycotic solution (Gibco). Myotube differentiation was induced by incubation in DMEM (Gibco) supplemented with 5% horse serum (Gibco) for 7 days. This protocol produces highly differentiated myotubes with distinctive gene expression profiles, and morphological and metabolic phenotypes (Torres-Velarde et al., 2021). Myotube differentiation was confirmed by microscopy (Fig. 3A). Myotubes were treated with the synthetic glucocorticoid dexamethasone (BioVision, Milpitas, CA, USA) at three ascending concentrations (0.1, 1.0 and 100 µmol l^−1^) for 6, 12 or 48 h. Dexamethasone induces a prolonged GR-mediated response while minimizing activation of the mineralocorticoid receptor in various cells, including elephant seal myotubes (Torres-Velarde et al., 2021). DMSO was used as a vehicle control. Cells were collected for DNA extraction after treatment and analyzed for global DNA methylation using a colorimetric Global DNA Methylation Assay kit (Abcam).

### Re-analysis of previously generated RNA-seq data

RNA-seq data from elephant seal myotubes treated with 100 µmol l^−1^ dexamethasone for 48 h (Torres-Velarde et al., 2021) were re-analyzed using our previously published protocol with minor modifications (Allen et al., 2024; Torres-Velarde et al., 2021). Briefly, total RNA was extracted using TRIzol (Invitrogen). RNA integrity was measured using a Bioanalyzer (Agilent Technologies, Santa Clara, CA, USA). RNA with RIN>9.5 was used to prepare cDNA libraries from poly(A)-captured mRNA using a KAPA RNA HyperPrep Kit (cat. no. KK8541, Roche, Basel, Switzerland) with TruSeq adapters from three replicates per treatment. A total of 25 mol l^−1^ reads per sample were sequenced on a NovaSeq platform (Illumina, San Diego, CA, USA) at the UC Berkeley Functional Genomics and Vincent J. Coates Genomics Sequencing Laboratories. Reads were mapped to the elephant seal genome (https://www.dnazoo.org/assemblies/mirounga_angustirostris) annotated in Torres-Velarde et al. (2021). Unannotated genes were manually curated and annotated using the latest version of the human genome reference (Ensembl GRCh38.p14). A BLAST search was performed against the human genome to identify homologous sequences, and Ensembl gene IDs were converted to gene symbols using the BioMart tool. Transcript levels were quantified using RSEM 1.3.1 (Li and Dewey, 2011). Genes differentially expressed between control and 100 µmol l^−1^ dexamethasone treatments were identified at a false discovery rate (FDR) of 5% using EBSeq (Leng et al., 2013). Over-representation analysis with gene ontology (GO) biological processes, KEEG and Reactome databases were conducted using the Webgestalt tool (Elizarraras et al., 2024) to identify enriched pathways in upregulated genes (FDR<0.05). Cis-regulatory element analysis was performed using iRegulon in Cytoscape at FDR<0.001 to identify the top three transcription factors acting on upregulated genes (Janky et al., 2014).

### Statistical analyses

Sample sizes were defined based on our previous work with this species (Ensminger et al., 2021; Torres-Velarde et al., 2021; Vázquez-Medina et al., 2010, 2013, 2011a,b). No samples were excluded from the analysis. Male and female animals were randomly allocated to each experimental group. Normality and homoscedasticity were assessed using D'Agostino–Pearson and Kolmogorov–Smirnov tests. Group differences were evaluated using ANOVA or Kruskal–Wallis (KW) tests, with Fisher's or Dunn's *post hoc* corrections, based on the mean of triplicate measurements per biological sample (individual seal or cell line). The relationship between global DNA methylation and cortisol was examined using Pearson correlation and Deming regression linear models. Differences were considered significant at *P*<0.05. Data are presented as means±s.d.

## RESULTS

### Prolonged fasting induces reversible blood DNA methylation in northern elephant seals

We measured the effects of prolonged fasting on plasma cortisol and global blood DNA methylation in northern elephant seals. Mean methylation values were 10 times higher in late than in early fasting seals and returned to early-fasting levels in post-foraging animals (KW=10.24, *P*=0.0015; Fig. 1B; Table S1). These data show that prolonged fasting alters DNA methylation profiles transiently in northern elephant seal blood. Whereas plasma cortisol levels were not statistically higher in late- than in early-fasting seals (*F*=5.206, *P*=0.0608), they were significantly lower in post-foraging than in late-fasting animals (*F*=5.206, *P*=0.0246) (Fig. 1C). Moreover, we detected a statistically significant positive correlation between plasma cortisol and global blood DNA methylation levels (*r*=0.554, *P*=0.0255), suggesting that blood DNA methylation increases with cortisol throughout the fasting period (Fig. 1D).

### The putative promoter region in the elephant seal GR gene contains one CpG island, which is not methylated in response to prolonged fasting in northern elephant seal blood

**Fig. 2.**
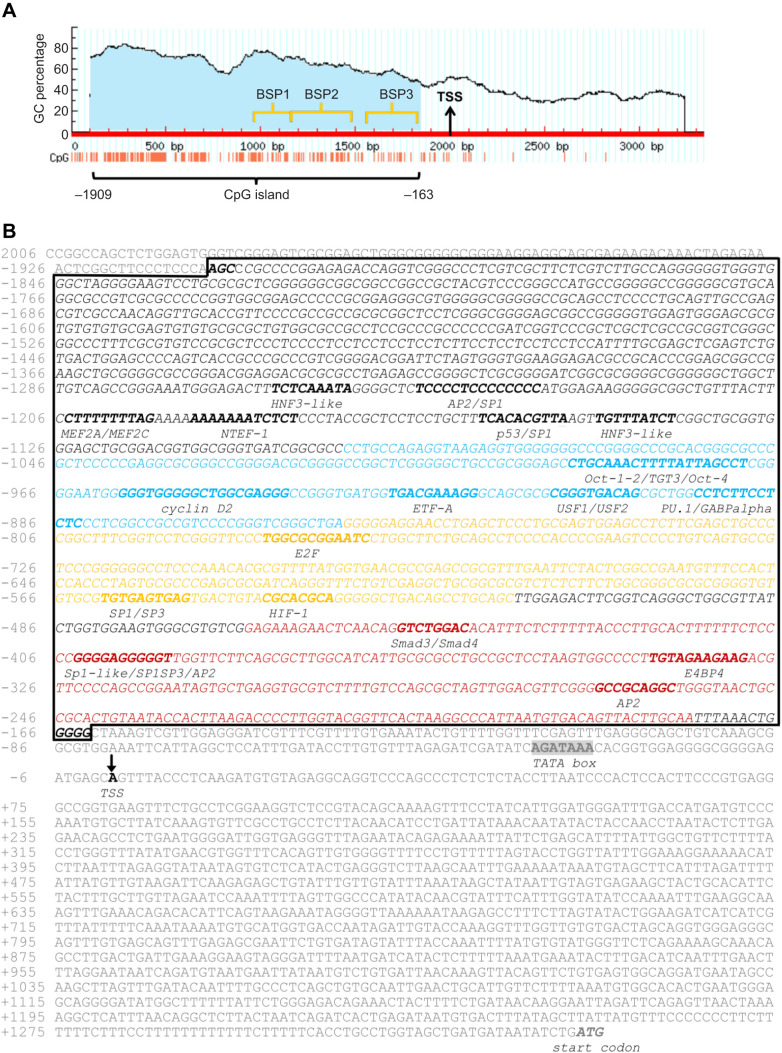
**CpG islands and binding motifs in the northern elephant seal glucocorticoid receptor (GR) gene.** (A) Identification of the CpG island (blue-shaded area) in the putative promoter region of the northern elephant seal GR gene. The CpG island was identified between nucleotide position −1908 and −162 (1747 bp length) upstream of the transcription start site (TSS). (B) Annotation of possible transcription factor binding motifs and other regulatory elements (bold) in the sequenced region of the northern elephant seal GR promoter (italic). Nucleotides in blue correspond to the section of DNA amplified by the primer pair MA-GR-BSP1; sequences in yellow correspond to the section of DNA amplified by the primer pair MA-GR-BSP2; sequences in red correspond to the section of DNA amplified by the primer pair MA-GR-BSP3. Primers were designed to amplify specific regions of the CpG island in the northern elephant seal GR promoter for methylation status characterization. The black box indicates the CpG island region. The TATA box (shaded gray), TSS (black arrow) and start codon (ATG; bold gray) are indicated. Analyses were conducted using Softberry NSITE software (http://www.softberry.com/berry.phtml?topic=nsite&group=programs&subgroup=promoter&advanced=on).

We searched for CpG islands in the elephant seal GR gene and looked for changes in GR methylation in this region in response to fasting in northern elephant seal blood. We detected one CpG island within the 3338 bp region of the elephant seal GR promoter (Fig. 2A). This CpG island is 1747 bp long, located between nucleotide positions −1909 and −163, upstream of the transcription start site (Fig. 2A). We then annotated transcription factor binding motifs and other regulatory elements in the sequenced region of the elephant seal GR promoter (Fig. 2B). The region of the GR promoter amplified by the BSP1 primer pair contained five regulatory element binding motifs, including binding sites for Oct-1-2-4/TGT3, cyclin D2, ETF-A, USF1/USF2 and PU.1/GABPα transcription factors. The GR promoter region amplified by the BSP2 primer pair contained three regulatory element binding motifs, including binding sites for E2F, SP1-3 and HIF-1 transcription factors. The region of the GR promoter amplified by the BSP3 primer pair contained four regulatory element binding motifs, including sites for Smad3/Smad4, SP1, E4BP4 and AP2 transcription factors (Fig. 2B). All cytosines within CpG sites in this GR promoter region were sequenced as thymines (T), suggesting most DNA molecules at this site were not methylated (early-fasting, late-fasting or post-foraging) (Figs S1–S3). These data show that in this region of the GR promoter, methylation does not change in response to prolonged fasting in blood cells, suggesting that if other transcription factors regulate the expression of the GR or other nearby genes, such regulation is likely not affected by methylation, at least in blood cells.

### Treatment with synthetic glucocorticoids decreases DNA methylation while upregulating metabolic and pro-survival genes in northern elephant seal myotubes

**Fig. 3.**
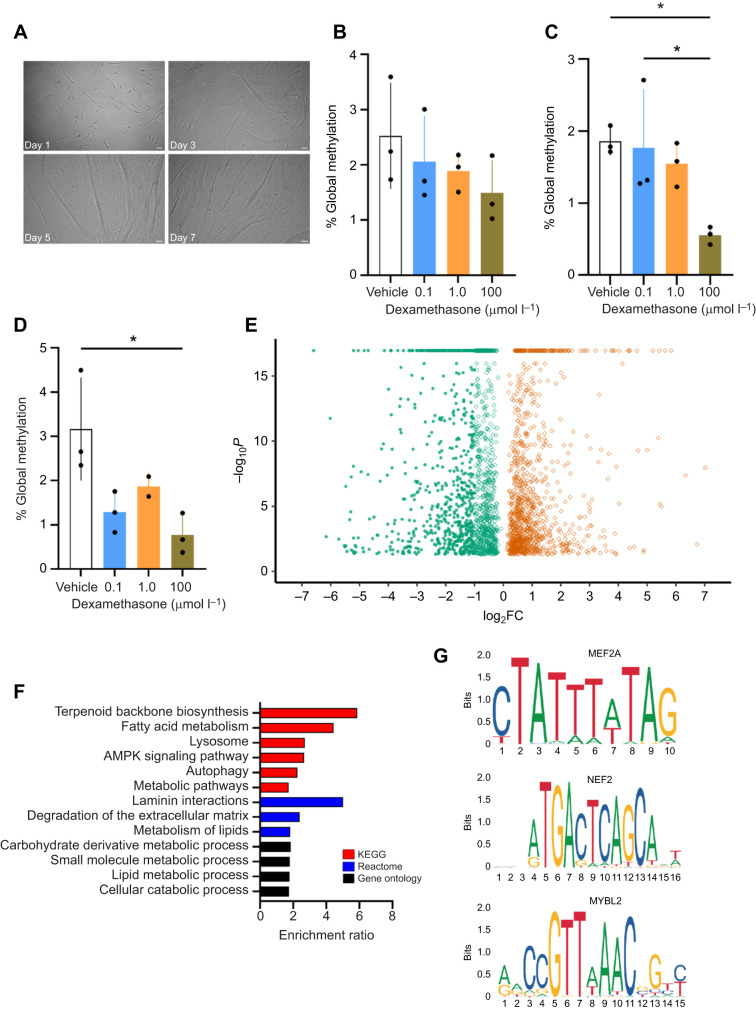
**Treatment with glucocorticoids decreases global methylation in northern elephant seal myotubes**. (A) Northern elephant seal myoblasts differentiating into myotubes. (B–D) DNA methylation in cells treated with ascending concentrations of dexamethasone (0.1, 1.0 and 100 µmol l^−1^) for (B) 6 h, (C) 12 h or (D) 48 h. Data are means±s.d. Asterisks indicate significance (ANOVA; *P<0.05). (E) Volcano plot showing differentially expressed genes (log_2_ fold-change, log_2_FC) in northern elephant seal myotubes treated with 100 µmol l^−1^ dexamethasone for 48 h (FDR<0.05). (F) Over-representation analysis (ORA) of upregulated differentially expressed genes (FDR<0.05). (G) Top enriched motifs identified by cis-regulatory element analysis of RNA-seq data. Sequence logos were retrieved from the Jaspar 2020 database (https://jaspar2020.genereg.net/). MEF2A, myocyte-specific enhancer factor 2A; NFE2, nuclear factor, erythroid 2; MYBL2, Myb-related protein B.

We tested whether exogenous treatment with the synthetic glucocorticoid dexamethasone, which induces potent, GR-specific signaling, alters global DNA methylation in northern elephant seal myotubes in primary culture. Treatment with ascending dexamethasone concentrations for 12 and 48 h, but not 6 h, decreased global methylation only at the highest dose (100 µmol l^−1^; Fig. 3B–D). These data suggest that northern elephant seal myotubes retain the capacity to respond to sustained high levels of glucocorticoids, potentially by regulating gene transcription. Hence, we re-analyzed RNA-seq data previously generated from elephant seal myotubes treated with 100 µmol l^−1^ dexamethasone for 48 h (Torres-Velarde et al., 2021). As described in our previous study, 2488 genes were differentially expressed (FDR<0.05%) in cells treated with dexamethasone compared with control, including 1078 upregulated genes and 1410 downregulated genes (Fig. 3E). Of the 2488 differentially expressed genes, 2406 were annotated, including 1045 upregulated genes and 1361 downregulated genes. Notably, dexamethasone treatment decreased GR gene expression (NR3C1) by 60% (not shown), further suggesting that elephant seal GR gene expression is not regulated by methylation. DNA methylation generally suppresses gene expression (Boyle et al., 2008; Newell-Price et al., 2000), and our experiments show that dexamethasone treatment decreases DNA methylation in northern elephant seal myotubes. Therefore, we focused on differentially expressed genes upregulated by dexamethasone. Overrepresentation analyses for GO biological processes, KEGG and Reactome pathways showed strong signatures for genes involved in muscle remodeling, energy metabolism and cell survival (Fig. 3F). We then analyzed cis-regulatory elements to identify enriched motifs for transcription factors acting on upregulated genes. Myocyte-specific enhancer factor 2A (MEF2A), nuclear factor, erythroid 2 (NFE2) and Myb-related protein B (MYBL2) were the top three predicted transcription factors, with normalized enriched scores of 5.069 (MEF2A), 5.054 (NFE2) and 4.618 (MYBL2) (Fig. 3G). MEF2 regulates skeletal muscle cell differentiation (Black and Olson, 1998; Edmondson et al., 1994) and cell survival via p38 MAPK signaling and interaction with STAT transcription factors (Moustafa et al., 2023). NFE2 is a known regulator of skeletal muscle (Llano-Diez et al., 2011), which interacts with CREB-binding protein (Yao et al., 2018), regulating transcription factors and genes responsible for lipogenesis and gluconeogenesis (Hung et al., 2001). NEF2 is also crucial for regulating antioxidant gene expression through AP1 binding sites (Shyu et al., 2005). MYBL2 regulates cell cycle progression, survival and differentiation via transcriptional regulation and direct interaction with serine-threonine kinase receptor-associated protein (STRAP), which suppresses TGF-β signaling (Musa et al., 2017). Hence, our data suggest that the observed decrease in DNA methylation in seal myotubes treated with dexamethasone is associated with a pro-survival transcriptional response that helps these cells cope with sustained glucocorticoid elevations (Torres-Velarde et al., 2021).

## DISCUSSION

DNA methylation is an adaptive mechanism that regulates gene expression in response to environmental stimuli (Reik, 2007). Here, we show that prolonged fasting transiently increases genome-wide blood DNA methylation in northern elephant seals in parallel with increasing plasma cortisol. Interestingly, our results also show that sustained exposure to the synthetic glucocorticoid dexamethasone decreases DNA methylation in cultured northern elephant seal muscle cells while upregulating the expression of metabolic and pro-survival genes. Hence, DNA methylation is potentially a cell type-specific strategy to fine-tune gene expression during energetically challenging conditions in northern elephant seals. Previous work showed that fasting elephant seals experience substantial systemic physiological adjustments. However, such adjustments are largely tissue specific, especially in seal pups, which simultaneously undergo fasting and postnatal development (Viscarra et al., 2013).

Previous studies also show that cyclical patterns in DNA methylation are a transient response to environmental stress (Alvarado et al., 2015; Coussement et al., 2023; Kangaspeska et al., 2008; Lin et al., 2020; Secco et al., 2015; Srere et al., 1992), supporting our conclusion that fasting-induced blood DNA methylation in northern elephant seals is a plastic mechanism to modify gene expression in response to environmental cues. Along with epigenetic changes in white blood cells, we detected changes in circulating cortisol in fasting seals. Post-weaning fasting increases plasma cortisol in elephant seal pups (Ortiz et al., 2003, 2001; Viscarra et al., 2011), likely contributing to lipid oxidation and endogenous glucose production (Champagne et al., 2013, 2005, 2012b; Rea and Costa, 1992; Viscarra et al., 2012). Hence, our results suggest that concerted systemic changes in plasma cortisol and blood DNA methylation may contribute to sustaining metabolic functions in fasting northern elephant seals.

While measuring global methylation shows overall changes over time, it does not provide details on specific methylated genes. Hence, we used bisulfite conversion, PCR amplification and Sanger sequencing (Zhang et al., 2009) to construct a specific methylation profile for the GR promoter, which regulates the neuroendocrine stress response to prolonged fasting and mediates metabolic adaptation in elephant seal muscle cells (Ensminger et al., 2021; Torres-Velarde et al., 2021). We sequenced regions within the CpG island of the GR promoter with three sets of BSP primers but did not find changes in blood GR methylation in fasting seal blood. It is important to note that the absence of specific methylation sites at the GR promoter measured by Sanger sequencing likely reflects the consensus of the most abundant DNA molecules. We do not rule out the possibility that a smaller fraction of DNA may be methylated, but this could not be detected with our method. High-throughput sequencing is recommended to quantify partial differences in DNA methylation, whereas further research is needed to assess the biological relevance of low-level changes in site-specific DNA methylation.

We also found that while sustained exposure to exogenous glucocorticoids decreases DNA methylation and upregulates pro-survival genes in northern elephant seal muscle cells, it suppresses GR gene expression, further suggesting that either the GR gene is not regulated by methylation or that a negative feedback mechanism suppresses GR expression during glucocorticoid elevations (Torres-Velarde et al., 2021). Of note, we did not evaluate other epigenetic mechanisms that might be involved in regulating GR expression, such as histone modifications (McCaw et al., 2020; Turner, 2002) or microRNAs (De Falco et al., 2023; Penso-Dolfin et al., 2020). Furthermore, while treatment with exogenous synthetic glucocorticoids is a powerful tool for inducing GR signaling in cultured cells, it may not accurately recapitulate fasting-induced increases in circulating cortisol. Hence, more *in vivo* studies are needed that directly evaluate epigenetic changes in complex tissues under natural fasting conditions.

Analysis of the sequenced region of the elephant seal GR promoter identified several binding sites for transcription factors that regulate chromatin remodeling (Kuznetsova et al., 2015; McDowell et al., 2018), cellular proliferation (Gay et al., 2016), the immune response (Adcock, 2001) and oxidant stress (You et al., 2009). Elephant seals are adapted to cope with oxidant stress induced by prolonged fasting and extended breath-hold diving (Crocker et al., 2016; Vázquez-Medina et al., 2012). The GR can interact with AP1 (Vesely et al., 2009), regulating the transcriptional response to oxidant stress (Zhou et al., 2001). We identified multiple AP1 binding sites within the sequenced region of the GR promoter, indicating that more than one AP1 subunit is involved in regulating GR expression. Our bisulfite sequencing results, however, showed that no binding sites were methylated in fasting northern elephant seals' blood cells. Furthermore, cis-regulatory element analysis of genes upregulated in northern elephant seal muscle cells treated with exogenous synthetic glucocorticoids identified NEF2, which regulates antioxidant gene expression through AP1 binding sites (Shyu et al., 2005), as an enriched transcription factor. AP1 has both positive and negative interactions with GR (Biddie et al., 2011; Yang-Yen et al., 1990), and is often activated in response to oxidant stress, but can also interact with GR during non-stress-mediated chromatin remodeling and mutually inhibitory protein–protein interactions. The lack of methylation in AP1 binding motifs despite the observed increases in global DNA methylation in seal blood suggests that GR and AP1 interact, potentially regulating antioxidant gene transcription during prolonged fasting (Crocker et al., 2016; Vázquez-Medina et al., 2010, 2013, 2011a).

DNA methylation is highly cell-type specific (Brandeis et al., 1993; Eden and Cedar, 1994) and plastic, with methylation or demethylation patterns regulating cellular differentiation throughout development (Carrió and Suelves, 2015). We observed opposite methylation patterns in white blood cells from fasting animals and cultured myotubes treated with exogenous synthetic glucocorticoids. During fasting, elephant seal weaned pups maintain muscle development without experiencing muscle atrophy, and elephant seal muscle cells use alternative metabolic pathways to support energy metabolism during sustained exposure to synthetic glucocorticoids (Torres-Velarde et al., 2021; Wright et al., 2020). The observed decrease in global methylation in response to sustained dexamethasone exposure in muscle cells and the over-representation of metabolic pathways in upregulated genes suggest that methylation might be an important mechanism to regulate genes involved in muscle development. Previous studies posited that DNA demethylation increases the expression of muscle proliferation genes (Brunk et al., 1996; Chiu and Blau, 1985). Further *in vivo* work is needed to understand how changes in methylation regulate gene expression in skeletal muscle at different time points during fasting.

In summary, we found that prolonged fasting increases global DNA methylation transiently in northern elephant seal blood, that blood DNA methylation profiles correlate with plasma cortisol levels, and that treatment with synthetic glucocorticoids decreases DNA methylation while upregulating pro-survival and metabolic genes in cultured northern elephant seal muscle cells. Hence, DNA methylation is a dynamic, potentially cell type-specific response that modulates gene expression in northern elephant seals. Future work on tissue- and gene-specific changes in DNA methylation patterns across the entire genome would contribute to our understanding of the dynamic role of DNA methylation in regulating gene expression in fasting seals. Using next-generation sequencing, such as whole-genome bisulfite sequencing (WGBS), would provide a more comprehensive view of the interactions between DNA methylation patterns, environmental factors and epigenetic modifications, increasing our understanding of gene expression regulation in fasting seals.

## Supplementary Material

10.1242/jexbio.250046_sup1 Supplementary information
