# Supplementary material for: Prolonged fasting and glucocorticoid exposure drive dynamic DNA methylation in northern elephant seals
Source: J Exp Biol. 2025 Jul 25;228(15):jeb250046. doi: 10.1242/jeb.250046 (PMC12319411; doi:10.1242/jeb.250046)
Supplement: Supplementary information [file jexbio-228-250046-s1.pdf]

A

Sequence name	mismatch (gap) / alignment length (% identity)	Me-CpG (%)	unconverted (% converted)	Methylation pattern (or reason for the exclusion ?)
Early-fasting (1)	7 (4) / 341 (97.9)	0 (0.0)	0/77 (100.0)	○○○○○○○○○○○○○○○○○○○○○○○○○○○○○○○○○
Early-fasting (2)	1 (1) / 339 (99.7)	0 (0.0)	0/77 (100.0)	○○○○○○○○○○○○○○○○○○○○○○○○○○○○○○○○○
Early-fasting (3)	0 (0) / 339 (100.0)	0 (0.0)	0/77 (100.0)	○○○○○○○○○○○○○○○○○○○○○○○○○○○○○○○○○
Early-fasting (4)	0 (0) / 339 (100.0)	0 (0.0)	0/77 (100.0)	○○○○○○○○○○○○○○○○○○○○○○○○○○○○○○○○○

B

Sequence name	mismatch (gap) / alignment length (% identity)	Me-CpG (%)	unconverted (% converted)	Methylation pattern (or reason for the exclusion ?)
Late-fasting (1)	0 (0) / 339 (100.0)	0 (0.0)	0/77 (100.0)	○○○○○○○○○○○○○○○○○○○○○○○○○○○○○○○○○
Late-fasting (2)	1 (0) / 339 (99.7)	0 (0.0)	0/77 (100.0)	○○○○○○○○○○○○○○○○○○○○○○○○○○○○○○○○○
Late-fasting (3)	2 (0) / 339 (99.4)	0 (0.0)	0/77 (100.0)	○○○○○○○○○○○○○○○○○○○○○○○○○○○○○○○○○
Late-fasting (4)	2 (1) / 339 (99.4)	0 (0.0)	0/77 (100.0)	○○○○○○○○○○○○○○○○○○○○○○○○○○○○○○○○○
Late-fasting (5)	1 (1) / 339 (99.7)	0 (0.0)	0/77 (100.0)	○○○○○○○○○○○○○○○○○○○○○○○○○○○○○○○×○

C

Sequence name	mismatch (gap) / alignment length (% identity)	Me-CpG (%)	unconverted (% converted)	Methylation pattern (or reason for the exclusion ?)
Post-fasting (1)	1 (1) / 339 (99.7)	0 (0.0)	0/77 (100.0)	○○○○○○○○○○○○○○○○○○○○○○○○○○○○○○○○○
Post-fasting (2)	0 (0) / 339 (100.0)	0 (0.0)	0/77 (100.0)	○○○○○○○○○○○○○○○○○○○○○○○○○○○○○○○○○
Post-fasting (3)	2 (1) / 340 (99.4)	0 (0.0)	0/77 (100.0)	○○○○○○○○○○○○○○○○○○○○○○○○○○○○○○○○○
Post-fasting (4)	1 (0) / 339 (99.7)	0 (0.0)	0/77 (100.0)	○○○○○○○○○○○○○○○○○○○○○○○○○○○○○○○○○
Post-fasting (5)	0 (0) / 339 (100.0)	0 (0.0)	0/77 (100.0)	○○○○○○○○○○○○○○○○○○○○○○○○○○○○○○○○○

Fig. S1. Methylation status in the GR promoter region (based on sequencing using BSP1 primers) in A) early fasting, B) late fasting, and C) post-foraging elephant seal pups. Filled-in (dark) circles indicate methylated CpG sites, and unfilled circles indicate unmethylated CpG sites. An "x" instead of a circle indicates that the CpG site was not sequenced. Data was compiled using QUMA software (http://quma.cdb.riken.jp/).

Journal of Experimental Biology • Supplementary information

A

Sequence name	mismatch (gap) / alignment length (% identity)	Me-CpG (%)	unconverted (% converted)	Methylation pattern (or reason for the exclusion ?)
Early-fasting (1)	3 (0) / 188 (98.4)	0 (0.0)	0/36 (100.0)	○○○○○○○○○○○○○○○○○○○○××○
Early-fasting (2)	4 (0) / 188 (97.9)	0 (0.0)	0/35 (100.0)	○○○○○○○○○○○○○○○○○○○○××○
Early-fasting (3)	2 (0) / 188 (98.9)	0 (0.0)	0/37 (100.0)	○○○○○○○○○○○○○○○○○○○○○×○
Early-fasting (4)	2 (0) / 188 (98.9)	0 (0.0)	0/36 (100.0)	○○○○○○○○○○○○○○○○○○○○○×○
Early-fasting (5)	1 (0) / 188 (99.5)	0 (0.0)	0/37 (100.0)	○○○○○○○○○○○○○○○○○○○○○○×

B

Sequence name	mismatch (gap) / alignment length (% identity)	Me-CpG (%)	unconverted (% converted)	Methylation pattern (or reason for the exclusion ?)
Late-fasting (1)	8 (4) / 188 (95.7)	0 (0.0)	0/33 (100.0)	×○○○○○○○○○○○○○○○○○○○××○
Late-fasting (2)	1 (0) / 188 (99.5)	0 (0.0)	0/36 (100.0)	○○○○○○○○○○○○○○○○○○○○○○○
Late-fasting (3)	2 (0) / 188 (98.9)	0 (0.0)	0/36 (100.0)	○○○○○○○○○○○○○○○○○○○○○×○
Late-fasting (4)	3 (0) / 188 (98.4)	0 (0.0)	0/36 (100.0)	○○○○○○○○○○○○○○○○○○○○○×○
Late-fasting (5)	2 (0) / 188 (98.9)	0 (0.0)	0/37 (100.0)	○○○○○○○○○○○○○○○○○○○○○×○

C

Sequence name	mismatch (gap) / alignment length (% identity)	Me-CpG (%)	unconverted (% converted)	Methylation pattern (or reason for the exclusion ?)
Post-fasting (1)	3 (0) / 188 (98.4)	0 (0.0)	0/35 (100.0)	○○○○○○○○○○○○○○○○○○○○○×○
Post-fasting (2)	2 (0) / 188 (98.9)	0 (0.0)	0/36 (100.0)	○○○○○○○○○○○○○○○○○○○○○×○
Post-fasting (3)	0 (0) / 188 (100.0)	0 (0.0)	0/37 (100.0)	○○○○○○○○○○○○○○○○○○○○○○○
Post-fasting (4)	4 (0) / 188 (97.9)	0 (0.0)	0/36 (100.0)	○○○○○○○○○○○○○○○○○○○○○××
Post-fasting (5)	0 (0) / 188 (100.0)	0 (0.0)	0/37 (100.0)	○○○○○○○○○○○○○○○○○○○○○○○

Fig. S2. Methylation status in the GR promoter region (based on sequencing using BSP2 primers) in A) early fasting, B) late fasting, and C) post-foraging elephant seal pups. Filled-in (dark) circles indicate methylated CpG sites, and unfilled circles indicate unmethylated CpG sites. An “x” instead of a circle indicates that the CpG site ot sequenced. Data was compiled using QUMA software (http://quma.cdb.riken.jp/).

Journal of Experimental Biology • Supplementary information

A

Sequence name	mismatch (gap) / alignment length (% identity)	Me-CpG (%)	unconverted (% converted)	Methylation pattern (or reason for the exclusion ?)
Early-fasting (1)	1 (1) / 222 (99.5)	0 (0.0)	0/49 (100.0)	○○○○○○○○○○○○○
Early-fasting (2)	1 (1) / 222 (99.5)	0 (0.0)	0/49 (100.0)	○○○○○○○○○○○○○
Early-fasting (3)	1 (1) / 222 (99.5)	0 (0.0)	0/49 (100.0)	○○○○○○○○○○○○○
Early-fasting (4)	1 (1) / 222 (99.5)	0 (0.0)	0/49 (100.0)	○○○○○○○○○○○○○
Early-fasting (5)	1 (1) / 222 (99.5)	0 (0.0)	0/49 (100.0)	○○○○○○○○○○○○○

B

Sequence name	mismatch (gap) / alignment length (% identity)	Me-CpG (%)	unconverted (% converted)	Methylation pattern (or reason for the exclusion ?)
Late-fasting (1)	1 (1) / 222 (99.5)	0 (0.0)	0/49 (100.0)	○○○○○○○○○○○○○
Late-fasting (2)	1 (1) / 222 (99.5)	0 (0.0)	0/49 (100.0)	○○○○○○○○○○○○○
Late-fasting (3)	1 (1) / 222 (99.5)	0 (0.0)	0/49 (100.0)	○○○○○○○○○○○○○
Late-fasting (4)	1 (1) / 222 (99.5)	0 (0.0)	0/49 (100.0)	○○○○○○○○○○○○○
Late-fasting (5)	1 (1) / 222 (99.5)	0 (0.0)	0/49 (100.0)	○○○○○○○○○○○○○

C

Sequence name	mismatch (gap) / alignment length (% identity)	Me-CpG (%)	unconverted (% converted)	Methylation pattern (or reason for the exclusion ?)
Post-fasting (1)	1 (1) / 222 (99.5)	0 (0.0)	0/49 (100.0)	○○○○○○○○○○○○○
Post-fasting (2)	1 (1) / 222 (99.5)	0 (0.0)	0/49 (100.0)	○○○○○○○○○○○○○
Post-fasting (3)	1 (1) / 222 (99.5)	0 (0.0)	0/49 (100.0)	○○○○○○○○○○○○○
Post-fasting (4)	1 (1) / 222 (99.5)	0 (0.0)	0/49 (100.0)	○○○○○○○○○○○○○
Post-fasting (5)	1 (1) / 222 (99.5)	0 (0.0)	0/49 (100.0)	○○○○○○○○○○○○○

Fig. S3. Methylation status in the GR promoter region (based on sequencing using BSP3 primers) in A) early fasting, B) late fasting, and C) post-foraging elephant seal pups. Filled-in (dark) circles indicate methylated CpG sites, and unfilled circles indicate unmethylated CpG sites. An “x” instead of a circle indicates that the CpG site was not sequenced. Data was compiled using QUMA software (http://quma.cdb.riken.jp/).

Journal of Experimental Biology • Supplementary information

Table S1. Raw methylation data.

Early-fasting pups		
Sample	***Avg OD***	***% meth***
MA19-01	*0.2765*	*0.0698683*
MA19-02	*0.4395*	*0.09464128*
MA19-03	*0.4215*	*0.0915221*
MA19-04	*0.8245*	*0.20533107*
MA19-05	*0.469*	*0.09998482*
MA19-06	*1.168*	*0.3674003*
Late-fasting pups		
Sample	***Avg OD***	***% meth***
MA19-10	*1.046*	*0.29274625*
MA19-11	*1.539*	*1.93736146*
MA19-12	*1.283*	*1.8396459*
MA19-13	*1.846*	*2.03505365*
MA19-14	*0.677*	*1.49628588*
MA19-15	*0.6345*	*1.54262287*
Post-foraging pups		
Sample	***Avg OD***	***% meth***
MA19-19	*0.3835*	*0.08527063*
MA19-20	*1.0215*	*0.27969252*
MA19-21	*0.8565*	*0.20571372*
MA19-22	*1.0295*	*0.28388967*
MA19-23	*1.0715*	*0.30698024*
Dexamethasone treatment		
	Avg OD	***% meth***
6hr/0.1uM/1	0.5205	3.006013759
6hr/0.1uM/2	0.419	1.704477827
6hr/0.1uM/3	0.3905	1.453467278
6hr/1uM/1	0.444	1.960112122
6hr/1uM/2	0.397	1.507247403
6hr/1uM/3	0.463	2.179741432
6hr/100uM/1	0.369	1.28887927
6hr/100uM/2	0.328	1.024899691

Journal of Experimental Biology • Supplementary information

6hr/100uM/3	0.462	2.167591288
6hr/cont/1	0.5525	3.594790616
6hr/cont/2	0.468	2.241521408
6hr/cont/3	0.422	1.73330146
12hr/0.1uM/1	0.502	2.710695225
12hr/0.1uM/2	0.3665	1.270993363
12hr/0.1uM/3	0.373	1.318021722
12hr/1uM/1	0.432	1.83294708
12hr/1uM/2	0.4055	1.580589142
12hr/1uM/3	0.36	1.225643023
12hr/100uM/1	0.223	0.569882362
12hr/100uM/2	0.251	0.666434577
12hr/100uM/3	0.1695	0.422580605
12hr/cont/1	0.4545	2.078598116
12hr/cont/2	0.42	1.71403205
12hr/cont/3	0.427	1.782428077
48hr/0.1uM/1	0.424	1.752787501
48hr/0.1uM/2	0.3675	1.278117747
48hr/0.1uM/3	0.2895	0.826455439
48hr/1uM/1	NA	NA
48hr/1uM/2	0.412	1.639072936
48hr/1uM/3	0.4555	2.09024942
48hr/100uM/1	0.3655	1.263908691
48hr/100uM/2	0.2495	0.660870168
48hr/100uM/3	0.1475	0.37368249
48hr/cont/1	0.498	2.650759712
48hr/cont/2	0.5925	4.495488522
48hr/cont/3	0.476	2.344032073
